# Variability of the Estimated Glomerular Filtration Rate in the First Year after Kidney Transplantation Is an Independent Risk Factor for Poor Renal Allograft Outcomes: A Retrospective Cohort Study

**DOI:** 10.1371/journal.pone.0168337

**Published:** 2016-12-14

**Authors:** Hoon Young Choi, Kyu Ha Huh, Jae Geun Lee, Mi Kyung Song, Myoung Soo Kim, Yu Seun Kim, Beom Seok Kim

**Affiliations:** 1 Department of Internal Medicine, Yonsei University College of Medicine, Seoul, Korea; 2 Department of Transplantation Surgery, Severance Hospital, Yonsei University Health System, Seoul, Korea; 3 The Research Institute for Transplantation, Yonsei University College of Medicine, Seoul, Korea; 4 Department of Biostatistics Collaboration Unit, Yonsei University College of Medicine, Seoul, Korea; University of Toledo, UNITED STATES

## Abstract

Renal function in the first year after kidney transplantation (KT) can predict long-term renal graft survival. This study investigated whether estimated glomerular filtration rate (eGFR) variability during the first year after KT is a risk factor for poor renal allograft outcomes. This retrospective cohort study included 3077 patients who underwent repeated eGFR measurements for 1 year after KT at Severance Hospital Transplantation Center between 1979 and 2012. The eGFR variability during the first year after KT was the predictor. The patients were divided into four quartile groups of eGFR variability according to the coefficient of variation for eGFR (eGFR-CV). We selected a cutoff of eGFR-CV for graft failure and performed the sensitivity analyses. The graft outcome was worse in the highest quartile group of eGFR variability than in the other groups among all patients (Q4: HR 1.631, 95% CI 1.278–2.081; p < 0.0001) and among patients without AR (Q4: HR 1.425, 95% CI 1.024–1.982; p = 0.0358) after adjusting for eGFR at 1 year after KT and other covariates. Additionally, all-cause mortality was higher in this highest quartile group than in the other groups among all patients but not among patients without AR. Higher eGFR-CVs than the cutoff were significantly associated with a high risk of graft failure among all patients (HR 1.670, 95% CI 1.395–2.000; p < 0.0001) and among patients without AR (HR 1.899, 95% CI 1.457–2.477; p < 0.0001) after fully adjusting for covariates. For all-cause mortality, a higher eGFR-CV was an independent risk factor among all patients but not among patients without AR after adjusting for covariates. eGFR variability in the first year after KT is an independent risk factor for poor renal allograft outcomes.

## Introduction

Renal function within the first year after kidney transplantation (KT) has been shown to be an important parameter that can influence long-term graft survival [1–5]. The estimated glomerular filtration rate (eGFR) has been commonly used to evaluate kidney function, and changes in the eGFR have been reported to be associated with the progression of kidney failure and negative outcomes [6–8]. However, the assessment of a single eGFR may not provide sufficient information on kidney function in different patients, who might have different levels of resilience to kidney issues [9]. Some studies have attempted to use eGFRs obtained repeatedly for modeling chronic kidney disease (CKD) progression with different statistical approaches, including modeling of nonlinear trajectories [9–12]. Moreover, a recent study measured the variability of kidney function by using repeated eGFR measurements and found that high variability was associated with an increased risk of death in stage 3–5 CKD patients [9, 13].

Although the graft attrition rate at 1 year after transplantation has improved, it has not translated into a proportionate improvement in long-term renal graft survival [14, 15]. In recent years, specific diseases, such as antibody-mediated rejection and de novo/recurrent glomerular diseases, have been identified as the primary causes of renal graft failure [15–18]. The inability to prevent or treat these specific diseases is likely associated with the insufficient improvement in long-term renal graft survival. One of the challenges for research in organ transplantation is the identification of markers that can sufficiently diagnose these specific diseases and that can be used as endpoints in clinical studies [15]. Additionally, many previous studies have attempted to identify markers of renal graft injury that can be used to improve long-term graft survival [15, 19–21].

The present study aimed to investigate whether eGFR variability during the first year after KT is a risk factor for poor renal allograft outcomes in a large population of kidney transplant recipients. It is important to realize the prognostic value of serial eGFR measurements during the first year after KT, as these can be easily and noninvasively obtained in kidney transplant recipients.

## Subjects and Methods

### Study population

This retrospective cohort study included adult patients who underwent KT at Severance Hospital Transplantation Center between 1979 and 2012. Patients who had poor early graft function (eGFR of <30 mL⋅min^-1^⋅1.73 m^-2^ at 1 month after KT) and those who did not have repeat eGFRs (every 3 months) during the first year after KT were excluded. A total of 3290 recipients who underwent KT were considered for inclusion in this study. Of these patients, 213 met the exclusion criteria and were not assessed. The immunosuppressive protocol was used as previously reported [5]. We also performed subgroup analysis for patients without an acute rejection (AR) episode during the first year after KT. This study was approved by the institutional review board of Severance Hospital, Yonsei University College of Medicine, Seoul, Korea, and informed consent was waived owing to the retrospective nature of the study.

### Clinical and pathological data

The clinical variables were donor and recipient age and sex, pre- and post-transplantation diabetes mellitus, hepatitis, dialysis duration before KT, human leukocyte antigen (HLA) mismatches, donor type (living related, living unrelated, and deceased), AR within 1 year of KT, the primary immunosuppressant, kidney function during the first year after KT, graft loss, and all-cause mortality. Kidney function was assessed with eGFRs that were calculated by using the CKD-Epidemiology Collaboration equation based on serum creatinine levels [22]. AR was defined according to the need for treatment, with or without biopsy confirmation. Graft failure was defined as patient death, or conversion to maintenance dialysis.

### eGFR variability

The mean eGFR and the eGFR variability were calculated as the mean and the coefficient of variation of serial eGFR measurements for each patient. The coefficient of variation of eGFR (eGFR-CV) was calculated as the ratio of the intra-individual standard deviation (SD) and mean in order to correct for a large SD. eGFRs were obtained every 3 months during the 1-year period after KT. The patients were divided into four quartile groups of eGFR variability according to the eGFR-CV and into four quartile groups of eGFR-mean.

### Statistical analysis

Data are presented as mean ± SD for continuous variables or frequency (percentage) for categorical variables. Statistical differences in the clinical characteristics among the four quartile groups according to eGFR-CV in the entire study group were determined using the analysis of variance test for continuous variables and the chi-square test for categorical variables.

The Kaplan-Meier method was used to evaluate graft failure and all-cause mortality in the four quartile groups by using the eGFR-mean and eGFR-CV. The estimated median time of graft failure and death in each group was also evaluated. The cumulative event rates in the groups were compared using the log-rank test. The method of Contal and O'Quigley was used to select the optimal cutoff of the eGFR-CV for graft failure [23]. Univariate and multivariable Cox regression analyses were performed to investigate the prediction of graft failure and all-cause mortality in the four quartile groups by using the eGFR-CV and the eGFR-CV cutoff. All analyses were performed using SAS version 9.2 (SAS Institute, Cary, NC, USA) and R package version 2.14.2 (R Foundation for Statistical Computing, Vienna, Austria). A p-value <0.05 was considered statistically significant.

## Results

**Fig 1:**
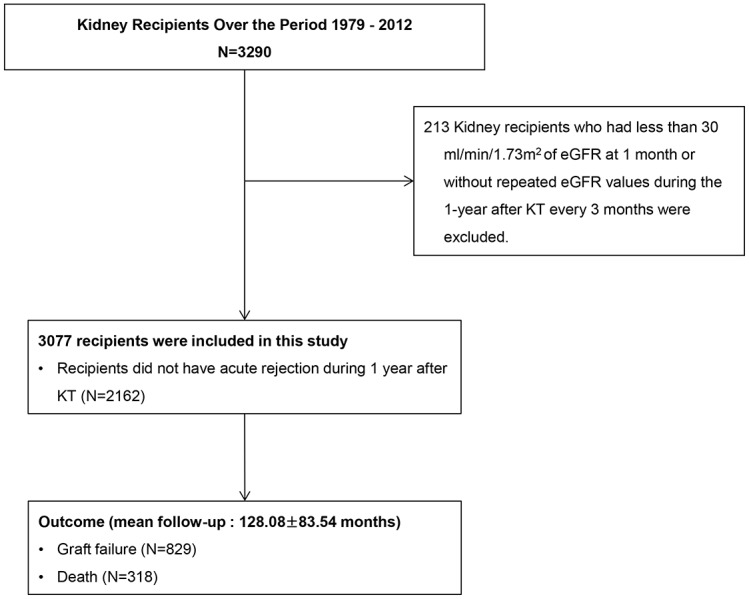
Algorithm used to define the study cohort.

A total of 3077 kidney transplant recipients were finally included in this study and were followed up for a mean of 128.08 ± 83.54 months (Fig 1).

The baseline characteristics of the patients in the four quartile groups of eGFR variability according to the eGFR-CV among all patients and among patients without AR during the first year after KT are presented in Table 1. AR events during the first year were more common and the eGFR at 1 year after KT was lower in the highest quartile group of eGFR variability than in the other groups.

**Table 1:** Baseline characteristics according to quartiles of eGFR variability in all patients and patients without AR.

Variables	All Patients				p-Value	Patients without AR				p-Value
	Q1	Q2	Q3	Q4		Q1	Q2	Q3	Q4	
**Age (years)**	40.15 ± 10.81	38.86 ± 10.90	39.86 ± 10.85	38.87 ± 11.10	0.0396	40.12 ± 11.06	39.45 ± 10.91	40.38 ± 10.91	39.93 ± 11.39	0.5538
**Sex (male, %)**	547 (70.49)	415 (66.72)	605 (66.56)	432 (59.59)	0.0001	337 (68.64)	406 (66.56)	321 (64.59)	315 (58.88)	0.0068
**DM [yes, n (%)]**	192 (24.74)	151 (24.28)	259 (28.49)	191 (26.34)	0.2101	106 (21.59)	150 (24.59)	125 (25.15)	137 (25.61)	0.4430
**DM type**					0.1418					0.6829
*No DM*	584 (75.26)	471 (75.72)	650 (71.51)	534 (73.66)		385 (78.41)	460 (75.41)	372 (74.85)	398 (74.39)	
*Pre-KT DM*	47 (6.06)	49 (7.88)	63 (6.93)	59 (8.14)		29 (5.91)	45 (7.38)	36 (7.24)	46 (8.60)	
*NODAT*	145 (18.69)	102 (16.40)	196 (21.56)	132 (18.21)		77 (15.68)	105 (17.21)	89 (17.91)	91 (17.01)	
**Hepatitis [yes, n(%)]**	67 (8.63)	50 (8.04)	81 (8.91)	90 (12.41)	0.0208	37 (7.54)	41 (6.72)	41 (8.25)	49 (9.16)	0.4738
**BMI at KT (kg/m**^**2**^**)**	21.78 ± 3.16	21.85 ± 3.25	21.77 ± 3.12	21.58 ± 3.17	0.5931	21.90 ± 3.23	21.91 ± 3.22	21.87 ± 3.16	21.64 ± 3.10	0.5770
**Donor age (years)**	37.69 ± 11.94	37.41 ± 11.78	36.94 ± 11.10	37.43 ± 11.73	0.6030	36.82 ± 11.67	38.17 ± 12.14	37.02 ± 10.98	37.33 ± 11.49	0.2194
**Donor sex (male, %)**	470 (60.57)	347 (55.79)	514 (56.55)	430 (59.31)	0.1998	289 (58.86)	350 (57.38)	268 (53.92)	310 (57.94)	0.4170
**Dialysis duration before KT (months)**	25.91 ± 38.99	21.56 ± 33.79	21.71 ± 33.46	27.74 ± 38.76	0.0016	26.80 ± 40.63	22.43 ± 33.80	20.45 ± 32.13	28.83 ± 39.57	0.0008
**HLA mismatch [yes, n (%)]**	689 (89.02)	540 (86.82)	788 (86.98)	644 (90.83)	0.0532	430 (87.93)	522 (85.57)	422 (85.25)	462 (87.50)	0.4891
**Number of HLA mismatches**	2.48 ± 1.30	2.38 ± 1.32	2.34 ± 1.32	2.49 ± 1.29	0.0605	2.50 ± 1.36	2.38 ± 1.33	2.32 ± 1.36	2.42 ± 1.38	0.1916
**Donor type [n (%)]**					<0.0001					0.0148
*LRD*	398 (51.29)	359 (57.72)	472 (51.93)	343 (47.31)		280 (57.03)	353 (57.87)	273 (54.93)	279 (52.15)	
*LURD*	288 (37.11)	222 (35.69)	384 (42.24)	300 (41.38)		152 (30.96)	200 (32.79)	189 (38.03)	189 (35.33)	
*Deceased*	90 (11.60)	41 (6.59)	53 (5.83)	82 (11.31)		59 (12.02)	57 (9.34)	35 (7.04)	67 (12.52)	
**AR during 1st year post-KT [yes, n (%)]**	101 (13.78)	119 (20.24)	199 (23.11)	289 (43.85)	<0.0001	-	-	-	-	-
**Main immunosuppressant [n (%)]**					<0.0001					<0.0001
*Aza*	9 (1.16)	11 (1.77)	29 (3.20)	54 (7.45)		6 (1.23)	6 (0.98)	15 (3.03)	30 (5.61)	
*CsA*	524 (67.70)	448 (72.03)	669 (73.76)	503 (69.38)		313 (64.01)	409 (67.05)	348 (70.30)	327 (61.12)	
*Tac*	241 (31.14)	163 (26.21)	209 (23.04)	168 (23.17)		170 (34.76)	195 (31.97)	132 (26.67)	178 (33.27)	
**eGFR (mL⋅min**^**-1**^**⋅1.73 m**^**-2**^**) at 1 year post-KT**	69.27 ± 17.68	68.69 ± 16.90	68.70 ± 17.65	62.15 ± 24.35	<0.0001	70.68 ± 17.15	69.77 ± 16.69	70.48 ± 17.22	66.26 ± 20.99	0.0003
**Graft survival (months)**	114.45 ± 84.70	127.59 ± 81.41	134.64 ± 85.95	116.12 ± 90.36	<0.0001	106.29 ± 85.19	123.98 ± 85.10	135.40 ± 90.76	123.08 ± 92.22	<0.0001

Data are expressed as mean ± SD or frequency (percentage); KT: kidney transplantation; DM: diabetes mellitus; Pre-KT DM: diabetes before KT; NODAT: new-onset diabetes after KT; BMI: body mass index; HLA: human leukocyte antigen; LRD: living related donor; LURD: living unrelated donor; AR: acute rejection; Aza: azathioprine; CsA: cyclosporine; Tac: tacrolimus; eGFR: estimated glomerular filtration rate.

**Fig 2:**
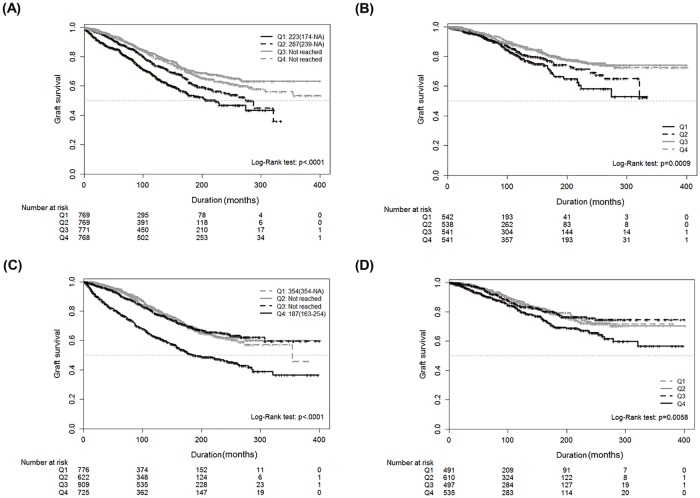
Kaplan–Meier curves for the median time of graft survival according to eGFR variability. *Upper panel*: Kaplan–Meier survival curves for graft failure by quartiles of the means of serially measured eGFR values in all patients (A) and in patients without AR (B) (black: quartile 1, black dotted: quartile 2, gray: quartile 3, gray dotted: quartile 4). *Lower panel*: Kaplan–Meier survival curves for graft failure by quartiles of the CV of serially measured eGFR values in all patients (C) and in patients without AR (D) (black: quartile 4, black dotted: quartile 3, gray: quartile 2, gray dotted: quartile 1). Black and gray lines indicate the quartile of eGFR-means or eGFR-CV values. eGFR: estimated glomerular filtration rate; AR: acute rejection; CV: coefficient of variation of eGFR.

In Kaplan–Meier survival curve analysis, graft survival among all patients and among patients without AR was lower in the lowest quartile group of the eGFR-mean than in the other groups (Fig 2A and 2B). Additionally, in Kaplan–Meier survival curve analysis, graft survival among all patients and among patients without AR was lower in the highest quartile group of eGFR variability than in the other groups (Fig 2C and 2D). The median graft failure time was 187 months in the lowest quartile group of eGFR variability (Fig 2C), whereas the median graft failure time was not obtained for the other quartile groups of eGFR variability (Fig 2D). Although the median graft failure time was not obtained in all quartile groups of eGFR variability among patients without AR, graft survival was found to be lower in the highest quartile group of eGFR variability than in the other groups.

In univariate Cox regression analysis for graft failure, the highest quartile group of eGFR variability was significantly associated with a high risk of graft failure among all patients (Q4: hazard ratio [HR] 2.077, 95% confidence interval [CI] 1.708–2.527; p < 0.0001) and among patients without AR (Q4: HR 1.537, 95% CI 1.117–2.114; p < 0.0001) (Table 2).

**Table 2:** Cox regression model for graft failure by quartiles of eGFR variability.

All patients			Patients without AR		
Variables	HR (95% CI)	p-Value	Variables	HR (95% CI)	p-Value
***Unadjusted***					
Q1 (≤0.09)	1(Ref)		Q1 (≤0.08)	1(Ref)	
Q2 (0.10–0.12)	1.015(0.806–1.277)	0.9021	Q2 (0.09–0.12)	1.037(0.743–1.447)	0.8329
Q3 (0.13–0.18)	1.050(0.854–1.292)	0.6411	Q3 (0.13–0.16)	1.025(0.727–1.444)	0.8888
Q4 (>0.18)	2.077(1.708–2.527)	<0.0001	Q4 (>0.16)	1.537(1.117–2.114)	0.0083
**Adjusted** ^a^			**Adjusted** ^b^		
Q1 (≤0.09)	1(Ref)		Q1 (≤0.08)	1(Ref)	
Q2 (0.10–0.12)	0.977(0.745–1.281)	0.8659	Q2 (0.09–0.12)	1.008(0.719–1.413)	0.9630
Q3 (0.13–0.18)	1.084(0.850–1.383)	0.5148	Q3 (0.13–0.16)	1.063(0.748–1.509)	0.7344
Q4 (>0.18)	1.631(1.278–2.081)	<0.0001	Q4 (>0.16)	1.425(1.024–1.982)	0.0358

^**a**:^ Adjusted by sex, donor age, donor type, HLA mismatches, DM, hepatitis, AR, eGFR at 1 year post-KT, and main immunosuppressant

^**b**:^ adjusted by sex, recipient and donor age, HLA mismatches, DM, hepatitis, eGFR at 1 year post-KT, and main immunosuppressant

HR: hazard ratio; CI: confidence interval; Ref: reference; AR: acute rejection; eGFR: estimated glomerular filtration rate; eGFR-CV: coefficient of variation of the estimated glomerular filtration rate; HLA: human leukocyte antigen; DM, diabetes mellitus; KT: kidney transplantation.

Multivariable Cox regression analysis for graft failure was performed after adjusting for covariates based on the univariate Cox regression analysis (S1 Table). Interestingly, in this analysis, the highest quartile group of eGFR variability was significantly associated with a high risk of graft failure among all patients (Q4: HR 1.631, 95% CI 1.278–2.081; p < 0.0001) and among patients without AR (Q4: HR 1.425, 95% CI 1.024–1.982; p = 0.0358) after adjusting for eGFR at 1 year after KT and other covariates (Table 2).

We then investigated the effect of eGFR variability on all-cause mortality among all patients and among patients without AR. In univariate Cox regression analysis for death, the highest quartile group of eGFR variability was significantly associated with a high risk of death among all patients (Q4: HR 2.077, 95% CI 1.708–2.527, p < 0.0001) and among patients without AR (Q4: HR 1.537, 95% CI 1.117–2.114; p = 0.0083) (Table 3). Multivariable Cox regression analysis for death was performed after adjusting for covariates based on the univariate Cox regression analysis (S2 Table). In this analysis, the highest quartile group of eGFR variability was significantly associated with a high risk of death among all patients (Q4: HR 1.738, 95% CI 1.036–2.916; p = 0.0363). However, there was no significant association with a high risk of death among patients without AR after adjusting for covariates (Table 3).

**Table 3:** Cox regression model for death by quartiles of eGFR variability.

All patients			Patients without AR		
Variables	HR (95% CI)	p-Value	Variables	HR (95% CI)	p-Value
***Unadjusted***			***Unadjusted***		
Q1 (≤0.09)	1(Ref)		Q1 (≤0.08)	1(Ref)	
Q2 (0.10–0.12)	1.015(0.806–1.277)	0.9021	Q2 (0.09–0.12)	1.037(0.743–1.447)	0.8329
Q3 (0.13–0.18)	1.050(0.854–1.292)	0.6411	Q3 (0.13–0.16)	1.025(0.727–1.444)	0.8888
Q4 (>0.18)	2.077(1.708–2.527)	<0.0001	Q4 (>0.16)	1.537(1.117–2.114)	0.0083
**Adjusted** ^a^			**Adjusted** ^b^		
Q1 (≤0.09)	1(Ref)		Q1 (≤0.08)	1(Ref)	
Q2 (0.10–0.12)	1.042(0.579–1.876)	0.8905	Q2 (0.09–0.12)	0.719(0.320–1.616)	0.4249
Q3 (0.13–0.18)	1.383(0.829–2.307)	0.2143	Q3 (0.13–0.16)	0.731(0.316–1.693)	0.4645
Q4 (>0.18)	1.738(1.036–2.916)	0.0363	Q4 (>0.16)	1.197(0.561–2.553)	0.6418

^**a**:^ Adjusted by sex, age, donor type, DM, hepatitis, AR, and main immunosuppressant

^**b**:^ adjusted by age, donor type, DM, hepatitis, and eGFR at 1 year post-KT

HR: hazard ratio; CI: confidence interval; Ref: reference; AR: acute rejection; eGFR: estimated glomerular filtration rate; DM, diabetes mellitus; KT: kidney transplantation.

### Sensitivity analyses

**Fig 3:**
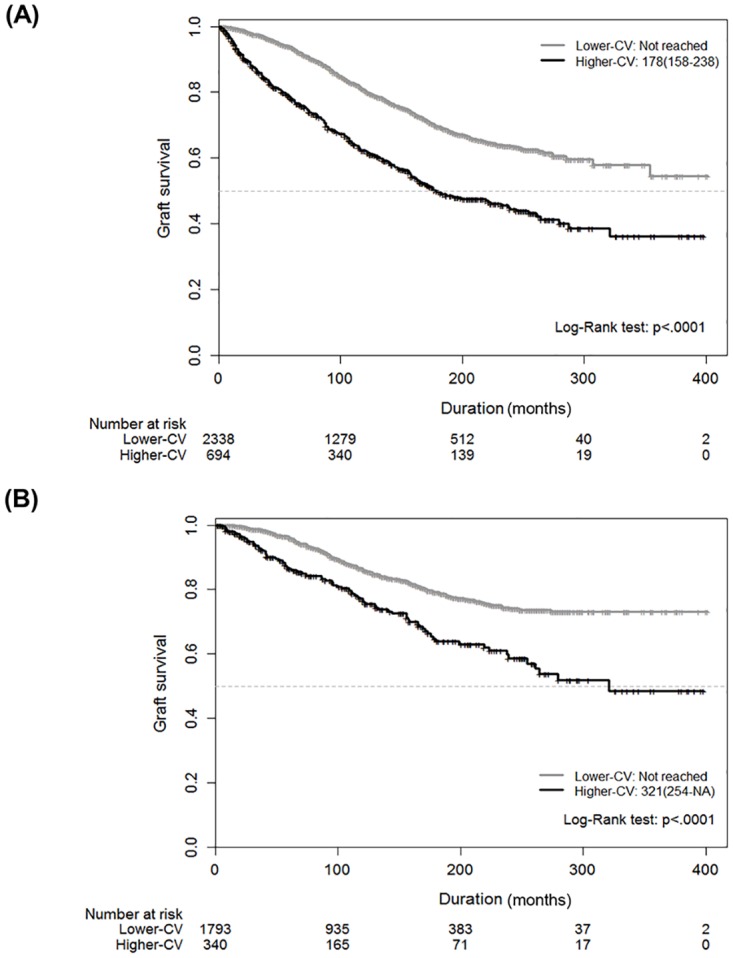
Kaplan–Meier survival curves for graft failure by using the cutoff of the eGFR-CV of serially measured eGFR values in all patients (A) and in patients without AR (B). Black and gray lines indicate the cutoff of eGFR-CV (black: higher-CV, gray: lower-CV). eGFR: estimated glomerular filtration rate; AR: acute rejection; CV: coefficient of variation of eGFR; higher-CV: higher values than the cutoff of eGFR-CV; lower-CV: lower values than the cutoff of eGFR-CV.

We used the method of Contal and O’Quigley to select a cutoff of eGFR-CV for graft failure [24], and we generated Kaplan–Meier survival curves with this cutoff. The cutoffs of 0.1832 among all patients and 0.1859 among patients without AR were selected using the abovementioned method. Kaplan–Meier survival curves with the cutoffs of eGFR-CV revealed that graft survival was significantly lower in the group with higher eGFR-CV than the cutoff than in the group with lower eGFR-CVs than the cutoff among all patients (Fig 3A) and among patients without AR (Fig 3B).

In univariate Cox regression analysis for graft failure according to the cutoff of eGFR-CV, a higher eGFR-CVs than the cutoff was associated with a high risk of graft failure among all patients and among patients without AR. Moreover, in multivariable Cox regression analysis, a higher eGFR-CVs than the cutoff was significantly associated with a high risk of graft failure among all patients (HR 1.670, 95% CI 1.395–2.000; p < 0.0001) and among patients without AR (HR 1.899, 95% CI 1.457–2.477; p < 0.0001) after fully adjusting for covariates, including eGFR at 1 year after KT (Table 4).

**Table 4:** Cox regression model for graft failure and death by using the cutoff of eGFR variability.

All patients			Patients without AR		
Variables	HR (95% CI)	p-Value	Variables	HR (95% CI)	p-Value
***Graft failure***					
** Unadjusted**	2.106 (1.824–2.431)	<0.0001	** Unadjusted**	1.996 (1.563–2.550)	<0.0001
** Adjusted**^a^	1.670 (1.395–2.000)	< .0001	** Adjusted** ^b^	1.899 (1.457–2.477)	<0.0001
***Death***					
** Unadjusted**	1.969 (1.555–2.492)	<0.0001	** Unadjusted**	1.822 (0.998–3.325)	0.0507
** Adjusted** ^c^	1.579 (1.104–2.257)	0.0122	** Adjusted** ^d^	1.522 (0.782–2.961)	0.2163

^**a**:^ Adjusted by sex, donor age, donor type, HLA mismatches, DM, hepatitis, AR, eGFR at 1 year post-KT, and main immunosuppressant

^**b**:^ adjusted by sex, recipient and donor age, HLA mismatches, DM, hepatitis, eGFR at 1 year post-KT, and main immunosuppressant

^**c**:^ adjusted by sex, age, donor type, DM, hepatitis, AR, and main immunosuppressant

^**d**:^ adjusted by age, donor type, DM, hepatitis, and eGFR at 1 year post-KT

eGFR: estimated glomerular filtration rate; AR: acute rejection; HR: hazard ratio; CI: confidence interval.

We then performed univariate and multivariable Cox regression analyses for death according to the cutoff of eGFR-CV. In the univariate Cox regression analysis, a higher eGFR-CVs than the cutoff was significantly associated with a high risk of death among all patients (HR 1.969, 95% CI 1.555–2.492; p < 0.0001); however, there was no significant association with a high risk of death among patients without AR (HR 1.822, 95% CI 0.998–3.325; p = 0.0507). In multivariable Cox regression analysis, a higher eGFR-CVs than the cutoff was significantly associated with a high risk of death among all patients after adjusting for covariates (HR 1.579, 95% CI 1.104–2.257; p = 0.0122); however, there was no significant association with a high risk of death among patients without AR after adjusting for covariates (Table 4).

## Discussion

We found that high eGFR variability in the first year after KT was associated with a high risk of graft failure. This risk was independent of well-known risk factors for kidney graft failure, such as donor type, HLA mismatches, and eGFR at 1 year after transplantation. We also noted a significant association between eGFR variability and all-cause mortality in all patients who underwent KT; however, this association was not significant in patients without AR episodes during the first year after KT.

To our knowledge, this is the first study to investigate the effects of eGFR variability on long-term graft survival in kidney transplant recipients. To date, single renal function parameters and changes in renal function, represented as delta creatinine between different time points during the first year after KT, have been reported as predictors for graft survival. In a previous large-scale retrospective survey, renal function assessed by using the serum creatinine levels at 1 year after transplantation was significantly associated with graft survival [1]. Interestingly, when renal function within the first year and clinical AR were included in the regression model for long-term graft survival, only the 1-year creatinine level and changes in creatinine values showed significance. A previous multicenter study has shown that serum creatinine measurements at 6 and 12 months can predict the 3-year graft survival [2]. In that study, a Cox regression analysis showed that serum creatinine levels at both 6 and 12 months after transplantation and the change in the serum creatinine level between these time points were important predictors of graft loss at 3 years after transplantation.

Recently, eGFR variability has been reported to indicate reduced kidney resilience to stimuli, and it might be a risk factor for kidney failure [9]. Some studies have attempted to use repeated measurements of the eGFR to model CKD progression by using different statistical approaches, including modeling of nonlinear trajectories [9–12]. In the present study, we also used repeated measurements of the eGFR in the first year after transplantation and calculated the eGFR variability. Multivariable Cox regression analysis revealed that a high eGFR variability was associated with a high risk of graft failure after fully adjusting for covariates that were well-known risk factors for graft failure, such as sex, age, donor type, HLA mismatches, diabetes mellitus, hepatitis, AR during the first year after KT, the primary immunosuppressant, and a single eGFR at 1 year after KT, among all patients and among patients without AR episodes in the first year.

Although new surrogate markers for late graft failure have been reported, renal function remains the best measure for predicting outcomes [15, 25]. However, it has been shown that measurement of kidney function at a fixed time after transplantation was not always predictive of the decline in function over time [25, 26]. Changes in the eGFR between 3 and 12 months after transplantation were shown to have a U-shaped relationship with graft failure, whereby both decreasing and increasing eGFRs were associated with poor outcomes [3]. This U-shaped relationship indicates that the eGFR variability itself may increase the risk of graft failure regardless of the slope of the eGFR trajectory in kidney transplant recipients.

Previous studies used eGFR variability in CKD patients as SDs, concurrent means, and slopes of eGFR progression [9, 27]. We measured eGFR variability as eGFR-CV, calculated by using the ratio of the intraindividual SD and the mean of repeated eGFR measurements during the first year after KT. As a normalized measure of variability, eGFR-CV was calculated as the ratio of the intrapersonal SD and mean to correct for large SDs because of high absolute eGFRs [28]. In the present study, we selected an eGFR-CV cutoff for graft failure by using the method of Contal and O’Quigley. This calculation can easily be used by clinicians for assessing renal allograft management. After KT, a clinician can assess a patient’s eGFR-CV in the first year, and the clinician should consider investigating acute kidney injury or other issues in patients with a high eGFR-CV, which indicates increased variability of renal function [9].

Several factors might explain the observed relationship between eGFR variability and renal graft failure. The variability of renal function may be related to loss of nephron mass, changes in renal plasma flow, or endothelial dysfunction. These factors have been shown to possibly be associated with death in kidney transplant recipients, and these factors can occur in CKD patients [27]. Furthermore, these factors can result in chronic allograft nephropathy (CAN), which is a major cause of late graft loss [29, 30]. Chronic rejection as a cause of graft loss was more common in kidney transplant recipients with a high eGFR variability in this study (data not shown), indicating that eGFR variability might be associated with an immune reaction, such as subclinical rejection, which could be related with CAN [31]. A previous report found that up to 27% of patients presented with subclinical rejection at 1 year after transplantation, without the clinician being aware of the condition. Additionally, the finding of normal and stable allograft function, represented by serum creatinine, has been observed despite biopsies showing lymphocytic infiltrates [32]. Accordingly, our results showing that eGFR variability is associated with graft loss independently with a single assessment of eGFR after KT are clinically important. This suggests that dynamic eGFR changes may provide appropriate predictive information for subclinical rejection and late graft loss together with traditional risk factors and renal function at a fixed time point.

A recent retrospective study reported that eGFR variability could predict death among patients with stage 3 CKD independent of previously well-known risk factors, such as diabetes, proteinuria, serum albumin, baseline eGFR, and eGFR slope [27]. Similarly, the present study revealed that high eGFR variability was associated with a significantly high risk of all-cause mortality among all kidney transplant recipients. However, eGFR variability and eGFR at 1 year after KT did not show any significant association with the risk of death among patients without AR. It is believed that other traditional risk factors for death, such as age, hypertension or diabetes status, and duration of pre-KT dialysis, are important in kidney transplant recipients without AR episodes in the first year after KT; however, the mechanisms are unclear [14].

The present study has several limitations. First, this was a retrospective study. Second, we did not evaluate the level of the calcineurin inhibitor, which can affect the kidney graft outcome. Third, the findings are limited by the inherent issues of serum creatinine, which is dependent on muscle mass, and generation and tubular secretion of creatinine [6, 16]. A previous study showed that low serum creatinine levels could result from muscle wasting caused by comorbid conditions in kidney transplant recipients [3]. Fourth, eGFR variability was assessed in patients who underwent KT at a single center. Therefore, to utilize this factor in other KT populations, the relative predictive strengths of various variability derivations should be tested. Finally, in this study, we could not investigate time-dependent covariates for outcomes in the transplant recipients. Kasiske et al. found that measures of change in chronic renal function, such as the duration of the first decline in inverse creatinine, declines in estimated creatinine clearance, and declines in the measured slope of 1/creatinine over time after KT, were better outcome predictors than baseline function and slope of inverse serum creatinine [26].

In conclusion, we found that eGFR variability in the first year after KT is an independent risk factor for poor renal allograft outcomes. Our results suggest that eGFR variability calculated by using serum creatinine may help predict long-term graft survival in kidney transplant recipients. Those who have higher eGFR variability should be monitoring carefully for preventing worse graft outcome.

## Supporting Information

S1 Table Univariate Cox regression analysis for graft failure. (PDF)

S2 Table Univariate Cox regression analysis for death. (PDF)
