# Supplementary material for: Variability of the Estimated Glomerular Filtration Rate in the First Year after Kidney Transplantation Is an Independent Risk Factor for Poor Renal Allograft Outcomes: A Retrospective Cohort Study
Source: PLoS One. 2016 Dec 14;11(12):e0168337. doi: 10.1371/journal.pone.0168337 (PMC5156409; doi:10.1371/journal.pone.0168337)
Supplement: S1 Table — (PDF) [file pone.0168337.s001.pdf]

S1 Table. Univariate Cox regression for graft failure

Variables	All patients		Patients without AR	
	HR (95% CI)	p-Value	HR (95% CI)	p-Value
**Age (years)**	0.997 (0.990-1.003)	0.33	0.983 (0.973-0.994)	0.002
**Sex**				
Male	1 (Ref)		1 (Ref)	
Female	0.749 (0.643-0.874)	<0.001	0.695 (0.545-0.886)	0.003
**Donor age (years)**	1.014 (1.008-1.020)	<0.001	1.022 (1.013-1.032)	<0.001
**Diabetes**				
No	1 (Ref)		1 (Ref)	
Pre-KT DM	1.255 (0.938-1.681)	0.13	1.035 (0.624-1.716)	0.89
NODAT	0.699 (0.588-0.833)	<0.001	0.516 (0.382-0.698)	<0.001
**Hepatitis**				
No	1 (Ref)		1 (Ref)	
Yes	1.751 (1.457-2.105)	<0.001	1.662 (1.231-2.244)	0.001
**AR within 1 year**				
No	1 (Ref)		-	-
Yes	2.364 (2.021-2.764)	<0.001	-	-
**Donor type**				
LRD	1 (Ref)		1 (Ref)	
LURD	1.167 (1.015-1.342)	0.03	0.996 (0.798-1.243)	0.97
Deceased	1.329 (0.933-1.893)	0.11	1.582 (0.957-2.614)	0.07
**HLA mismatches**				
No	1 (Ref)		1 (Ref)	
Yes	1.577 (1.230-2.023)	<0.001	1.704 (1.177-2.466)	0.005
**Main immunosuppressant**				
Aza	1 (Ref)		1 (Ref)	
CsA	0.541 (0.421-0.696)	<0.001	0.753 (0.488-1.164)	0.20
Tac	0.200 (0.134-0.300)	<0.001	0.416 (0.232-0.748)	0.003
**eGFR at 1 year post-KT**	0.981 (0.977-0.985)	<0.001	0.983 (0.977-0.990)	<0.001

KT: kidney transplantation; DM: diabetes mellitus; Pre-KT DM: diabetes before KT; NODAT: new-onset diabetes after KT; AR: acute rejection; LRD: living related donor; LURD: living unrelated donor; HLA: human leukocyte antigen; Aza: azathioprine; CsA: cyclosporine; Tac: tacrolimus; eGFR: estimated glomerular filtration rate; HR: hazard ratio; CI: confidence interval.
