# Supplementary material for: Variability of the Estimated Glomerular Filtration Rate in the First Year after Kidney Transplantation Is an Independent Risk Factor for Poor Renal Allograft Outcomes: A Retrospective Cohort Study
Source: PLoS One. 2016 Dec 14;11(12):e0168337. doi: 10.1371/journal.pone.0168337 (PMC5156409; doi:10.1371/journal.pone.0168337)
Supplement: S2 Table — (PDF) [file pone.0168337.s002.pdf]

**S2 Table. Univariate Cox regression for death**

| Variables | All patients | | Patients without AR | |
|---|---|---|---|---|
| | HR (95% CI) | p-Value | HR (95% CI) | p-Value |
| **Age (years)** | 1.050(1.039-1.061) | <0.001 | 1.075(1.050-1.101) | <0.001 |
| **Sex** | | | | |
| Male | 1 (Ref) | | 1 (Ref) | |
| Female | 0.746(0.582-0.956) | 0.02 | 0.867(0.505-1.488) | 0.6 |
| **Donor age (years)** | 1.005(0.995-1.015) | 0.3417 | 1.017(0.995-1.039) | 0.13 |
| **Diabetes** | | | | |
| No | 1 (Ref) | | 1 (Ref) | |
| Pre-KT DM | 2.364(1.625-3.438) | <0.001 | 3.756(1.925-7.332) | <0.001 |
| NODAT | 1.085(0.837-1.405) | 0.54 | 0.796(0.398-1.591) | 0.52 |
| **Hepatitis** | | | | |
| No | 1 (Ref) | | 1 (Ref) | |
| Yes | 2.598(1.995-3.383) | <0.001 | 2.559(1.362-4.806) | 0.004 |
| **AR within 1 year** | | | | |
| No | 1 (Ref) | | - | - |
| Yes | 3.979(2.874-5.509) | <0.001 | - | - |
| **Donor type** | | | | |
| LRD | 1 (Ref) | | 1 (Ref) | |
| LURD | 1.420(1.131-1.782) | 0.003 | 1.151(0.668-1.982) | 0.61 |
| Deceased | 1.613(0.956-2.723) | 0.07 | 3.448(1.615-7.361) | 0.001 |
| **HLA mismatches** | | | | |
| No | 1 (Ref) | | 1 (Ref) | |
| Yes | 1.460(0.988-2.157) | 0.06 | 1.894(0.759-4.726) | 0.17 |
| **Main immunosuppressant** | | | | |
| Aza | 1 (Ref) | | 1 (Ref) | |
| CsA | 0.327(0.233-0.458) | <0.001 | 1.121(0.267-4.701) | 0.88 |
| Tac | 0.149(0.085-0.261) | <0.001 | 1.535(0.341-6.921) | 0.58 |
| **eGFR at 1 year post-KT** | 0.994 (0.988-1.000) | 0.06 | 0.977 (0.961-0.994) | 0.01 |

KT: kidney transplantation; BMI: body mass index; AR: acute rejection, Pre-KT DM: diabetes before KT; NODAT: new-onset diabetes after KT; AR: acute rejection; LRD: living related donor; LURD: living unrelated donor; HLA: human leukocyte antigen; Aza: azathioprine; CsA: cyclosporine; Tac: tacrolimus; eGFR: estimated glomerular filtration rate; HR: hazard ratio; CI: confidence interval.
